# Characteristics of pain in patients with NMOSD and MOGAD: impact on mental health, sleep and quality of life

**DOI:** 10.1007/s10072-025-08277-6

**Published:** 2025-06-07

**Authors:** Emine Rabia Koc, Furkan Saridas, Mehmet Fatih Yetkin, Nuray Bilge, Yasemin Dinc, Emel Oguz Akarsu, Sarra Elhamida Lazrak, Sanja Gluscevic, Abdulkadir Tunc, Meral Seferoglu, Caner Baydar, Ali Ozhan Sıvacı, O. Faruk Turan, Guven Ozkaya

**Affiliations:** 1 Faculty of Medicine, Department of Neurology, Bursa Uludag University, Uludag, Bursa, Turkey; 2 Faculty of Medicine, Department of Neurology, Erciyes University, Kayseri, Turkey; 3 Faculty of Medicine, Department of Neurology, Atatürk University, Erzurum, Turkey; 4 Clinical Centre of Montenegro, Clinic for Neurology, Podgorica, Montenegro; 5 Faculty of Medicine, Department of Neurology, Sakarya University, Sakarya, Turkey; 6 Bursa High Specialization Training and Research Hospital, Department of Neurology, Bursa, Turkey; 7 Faculty of Medicine, Department of Neurology, Van Yüzüncü Yıl University, Van, Turkey; 8 Faculty of Medicine, Department of Biostatistics, Uludag University, Bursa, Turkey

**Keywords:** NMOSD, MOGAD, Neuropathic pain, Quality of life, Mental health, Pain management

## Abstract

Neuromyelitis optica spectrum disorders (NMOSD) and myelin oligodendrocyte glycoprotein-associated disease (MOGAD) are autoimmune disorders frequently accompanied by chronic, often neuropathic, pain, which significantly impacts the quality of life, sleep, and mental health. This study evaluated the incidence and characteristics of neuropathic pain in 106 patients with NMOSD or MOGAD and assessed its effects on mental health, sleep quality, and overall quality of life. Using clinical evaluation and MRI findings to localize lesions, pain classification revealed that chronic pain was more common in NMOSD patients (78.4%) than in MOGAD patients (52.7%), with a significant impact on both groups. Patients with MOGAD who experienced neuropathic pain reported notably poorer sleep quality and higher anxiety and depression levels. Pain severity was strongly associated with spinal cord lesion length and thoracic location, particularly in MOGAD patients. Current treatments provide insufficient pain relief, highlighting the need for more effective management strategies. This study emphasized that neuropathic pain substantially diminishes both physical and mental well-being in NMOSD and MOGAD patients, highlighting the importance of personalized pain management approaches to improve quality of life and mental health in these populations.

## Introduction

Neuromyelitis optica spectrum disorders (NMOSD) and myelin oligodendrocyte glycoprotein antibody-associated disease (MOGAD) are two distinct autoimmune central nervous system diseases, with overlapping clinical and radiological features but different pathophysiology and antibody profiles [1, 2].

Pain, a subjective experience influenced by biological, psychological, and social factors, is a prevalent symptom of NMOSD and MOGAD that significantly impacts patients’ sleep and quality of life [3–8].

Chronic pain is classified into nociceptive, neuropathic, and nociplastic types. Nociceptive pain occurs when mechanical or thermal stimuli or toxic substances activate nociceptors. Nociplastic pain is a change in the perception of pain without damage to the nervous system or tissue. Neuropathic pain arises from a lesion or disease impacting the somatosensory nervous system [9]. NMOSD patients often experience severe and difficult-to-treat neuropathic pain. Previous studies have shown that only a small percentage of patients take specific medications for pain, and these medications provide only moderate relief [7, 8, 10, 11]. In the literature, systematic studies on NMOSD and MOGAD-associated pain syndromes are scarce, and most existing studies include AQP4-IgG-positive or AQP4-IgG-negative NMOSD patients [7, 8, 12].

Here, we aimed to determine the demographic, clinical, and radiological predictors of chronic neuropathic pain in patients with NMOSD or MOGAD and to investigate the effects of pain on mental health, sleep, and quality of life.

## Materials and methods

This cross-sectional study, which was conducted from 2020 to 2024, included 106 patients from six centers identified through local electronic databases. Among these patients, 51 were AQP4-IgG positive, and 55 were MOG-IgG positive. Antibodies (MOG-IgG, AQP4-IgG) were tested via a commercial cell-binding kit (Euroimmun) during NMOSD or MOGAD diagnosis, following clinical and radiological justification. Patients were interviewed and examined to classify pain as neuropathic or nonneuropathic (nociceptive, nociplastic). The inclusion criteria required patients to be 18 years or older, be diagnosed with NMOSD according to the 2015 diagnostic criteria or with MOGAD based on international panel criteria [2, 13], and be in remission from inflammatory disease. All patients were enrolled during remission (no active relapse) to ensure that pain assessments reflected chronic baseline symptoms rather than acute relapse-related pain, thus avoiding confounding effects of active inflammation on pain, mood, or sleep [8]. The exclusion criterion was individuals with other chronic pain syndromes, such as diabetic neuropathy, severe radix compression, or CNS lesions causing neuropathic pain.

Neuropathic pain was diagnosed when the pain distribution was neuroanatomically plausible for a lesion in the somatosensory pathways, with a corresponding CNS lesion confirmed on MRI. Notably, we did not require the lesion to produce overt sensory deficits; as Finnerup et al. (2016) emphasizes, “sensory loss is not a prerequisite for a neuropathic pain condition” [14] and documentation of an anatomically appropriate (even asymptomatic) lesion was accepted as sufficient evidence of neuropathic pain [14]. Ethics approval was obtained from our University Ethics Committee (No: 2024-13/7), and all participants provided written informed consent.

**Fig. 1:**
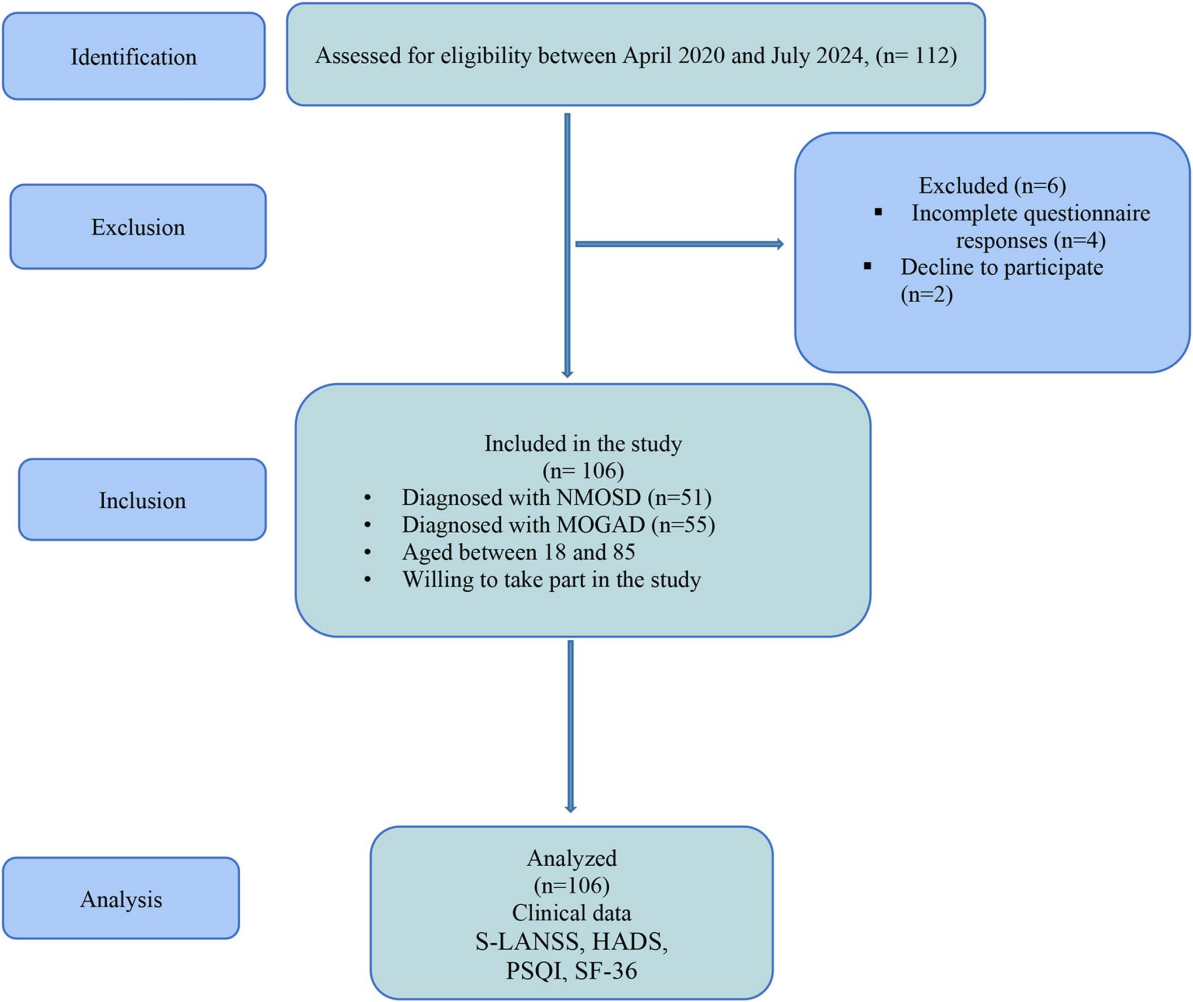
Study enrollment flow chart

Patients completed questionnaires in a designated room via the S-LANSS, NRS, HADS, PSQI, and SF-36 scales. Of the initial 112 participants, six were excluded because of incorrect diagnoses, incomplete responses, or withdrawals, leaving 106 patients for analysis. The study followed the STROBE checklist (Fig. 1).

### Clinical data and evaluation

The collected data included demographics, disease duration, relapse history (optic neuritis, myelitis), prior and current treatments, autoimmune comorbidities, EDSS disability scores, lesion location and length, and chronic pain presence or prophylaxis use. Pain characteristics and scores from the S-LANSS, NRS, SF-36, PSQI, and HADS were also recorded.

### Instruments

#### S-LANSS (self-reported Leeds assessment of neuropathic symptoms and signs scale)

This scale contains five items assessing pain symptoms and two items for self-administered sensory tests (allodynia and reduced pinprick sensation). The responses are binary (yes/no), with weighted scores predicting neuropathic pain. Scores range from 0 to 24, and scores ≥ 12 suggest likely neuropathic pain [15]. The Turkish adaptation was validated by Koc et al. [16]. In our study, neuropathic pain was clinically and radiologically confirmed, with the S-LANSS used as a supplementary tool.

### The numeric rating scale (NRS)

The NRS measures pain severity on a 0–10 scale, where 0 means no pain and 10 indicates the worst imaginable pain [17].

### Pittsburgh sleep quality index (PSQI)

The PSQI assesses sleep quality through 19 items scored from 0 to 21. Scores ≥ 5 indicate poor sleep quality [18]. Agargun et al.. validated the Turkish version [19].

### The hospital anxiety and depression scale (HADS)

The HADS screens for anxiety and depression and consists of 14 items rated 0–3. Scores ≥ 11 on either subscale suggest a clinically significant condition [20–22]. Aydemir et al.. adapted and validated the Turkish version [23].

### Short form-36 (SF-36)

The SF-36 evaluates physical and mental health across eight subscales, generating physical (PCS) and mental (MCS) component summary scores. Higher scores reflect better health status [24]. Kocyigit et al.. validated the Turkish version [25].

### Statistical analysis

Data distribution was evaluated with the Shapiro‒Wilk test. The results are presented as medians with interquartile ranges (IQRs) for continuous variables and as frequencies (percentages) for categorical variables. Normally distributed data were analyzed via independent samples t tests or one-way ANOVA, whereas nonnormally distributed data were assessed via the Kruskal‒Wallis and Mann‒Whitney U tests. The Bonferroni correction was applied for multiple comparisons. Categorical variables were compared via the Pearson chi-square test, Fisher’s exact test, and Fisher–Freeman–Halton test. Statistical significance was set at α = 0.05. Analyses were conducted with IBM SPSS 29.0.2.0 (IBM Corp., 2023).

## Results

### Demographic and clinical characteristics

The study population was divided into groups on the basis of the presence and type of pain (neuropathic or nonneuropathic). Chronic pain was detected in 78.4% of the AQP4-IgG positive NMOSD patients and 52.7% of the MOGAD patients. Among NMOSD patients, the female predominance was consistent across groups, without significant differences (*p* = 0.231). The median age ranged from 42 to 52 years, with no significant variation between groups (*p* = 0.171). The active smoking rates were similar across the groups (*p* = 0.965).

Among MOGAD patients, pain incidence and demographic characteristics were not significantly different (*p* = 0.624). The median age varied from 30.5 to 38 years across the subgroups (*p* = 0.092), and the smoking rates were comparable (*p* = 0.740).

### Disease duration, first clinical presentation, and number of attacks

Transverse myelitis was the most common first clinical presentation in the neuropathic pain group (57.7% in the NMOSD group and 57.9% in the MOGAD group). In MOGAD, transverse myelitis was more prevalent in the neuropathic pain group than in the nonneuropathic group (*p* = 0.043). NMOSD patients with neuropathic pain had significantly more attacks (median 2 vs. 1; *p* = 0.042).

### Lesion characteristics, length, and disability

Lesion localization was broadly similar across pain subgroups in both diseases. All patients with neuropathic pain had at least one identifiable CNS lesion relevant to the pain distribution. In both NMOSD and MOGAD, cervical and thoracic spinal cord lesions were the most common locations in patients with neuropathic pain. However, in MOGAD patients, those with neuropathic pain were significantly more likely to have a thoracic spinal cord lesion (52.6%) compared to MOGAD patients without neuropathic pain (15.4%, *p* = 0.020)​., Additionally, the median longest spinal cord lesion was greater in MOGAD patients with neuropathic pain (median 2 vertebral segments) than in those without neuropathic pain (median 0.5 segments, *p* = 0.007),. So, in MOGAD cohort, longer spinal cord lesions are associated with neuropathic pain. In the NMOSD cohort, spinal lesion length was high in all subgroups (median ~ 4 segments) and did not differ significantly by pain status (*p* = 0.892), as many AQP4-NMOSD patients had longitudinally extensive transverse myelitis regardless of pain presence​. EDSS disability scores were higher in the neuropathic pain groups for both NMOSD (median EDSS 3 vs. 1, *p* = 0.002) and MOGAD (median 2 vs. 0, *p* = 0.005) patients​, reflecting that patients with neuropathic pain had greater neurological impairment on follow-up. Consistently, EDSS at nadir was also significantly higher in those with neuropathic pain (especially in MOGAD, many neuropathic pain patients had a myelitis attack causing temporary severe disability). These findings suggest that more severe attacks and greater spinal involvement predispose patients to chronic neuropathic pain.

Lesion length analysis was refined by region according to the both subgroups in NMOSD and MOGAD cohort. Spinal cord lesions tended to be longer in AQP4-IgG-positive NMOSD (median longest spinal lesion 4 vertebral segments) than in MOGAD (2 segments), although this difference did not reach statistical significance (*p* = 0.15). In contrast, the degree of optic nerve involvement differed between groups: bilateral optic neuritis occurred more often in NMOSD (23.1%) than in MOGAD (5.3%), though this difference was not significant (*p* = 0.21). The median EDSS at last follow-up was 3 (2–3) in AQP4-IgG NMOSD patients and 2 (1–5) in MOGAD patients (*p* = 0.283), reflecting generally mild to moderate disability in both groups.

Notably, EDSS at nadir (peak disability during acute attacks) was higher in AQP4-IgG NMOSD patients (median 6, IQR 4–8) than in MOGAD patients (median 5, IQR 3–7), suggesting more severe acute attacks in NMOSD, although some MOGAD patients also experienced high nadir disability (e.g., during severe optic neuritis or myelitis (Table 1) Attack Treatment.

**Table 1:** Demographic, radiological, and clinical characteristics of patients with NMOSDand MOGAD

		NMOSD			*p* value within NMOSD	MOGAD			*p* value within MOGAD
		Without Pain	With Neuropathic Pain	With Nonneuropathic Pain		Without Pain	With Neuropathic Pain	With Nonneuropathic Pain	
Gender	Female	7 (63.6%)	22 (84.6%)	9 (64.3%)	0.231	16 (61.5%)	13 (68.4%)	5 (50%)	0.624
	Male	4 (36.4%)	4 (15.4%)	5 (35.7%)		10 (38.5%)	6 (31.6%)	5 (50%)	
Age (years)		42(30–46)	48.5(42–55)	52(41–55)	0.171	30.5(23–41)	38(27–46)	37(35–43)	0.092
Smoking	Active smoker	3 (27.3%)	7 (26.9%)	4 (28.6%)	0.965	9 (34.6%)	7 (36.8%)	6 (60%)	0.740
	Ex-smoker:	1 (9.1%)	5 (19.2%)	3 (21.4%)		6 (23.1%)	5 (26.3%)	1 (10%)	
	Never used	7 (63.6%)	14 (53.8%)	7 (50%)		11 (42.3%)	7 (36.8%)	3 (30%)	
Disease duration/year		4(1–6)	4(3–6)	3(2–5)	0.461	3(2–5)	4(3–6)	4.25(2–6)	0.293
First Clinical Presentation	Optic neuritis	6 (54.5%)	11 (42.3%)	8 (57.1%)	0.615	15 (57.7%)	10 (52.6%)	7 (70%)	0.665
	Transverse myelitis	5 (45.5%)	15 (57.7%)	4 (28.6%)	0.229	10(38.5%)^ab^	11 (57.9%)^b^	1 (10%)^a^	0.043
	area postrema	1 (9.1%)	1 (3.8%)	0 (0%)	0.460	2 (7.7%)	1 (5.3%)	0 (0%)	1.000
	brainstem	1 (9.1%)	4 (15.4%)	1 (7.1%)	0.855	1 (3.8%)	1 (5.3%)	2 (20%)	0.231
	cerebral	1 (9.1%)	0 (0%)	1 (7.1%)	0.235	1 (3.8%)	0 (0%)	0 (0%)	1.000
	others	0 (0%)	0 (0%)	1 (7.1%)	0.490	0 (0%)	0 (0%)	0 (0%)	-
Number of relapses		1(1–2)^a^	2(1–5)^b^	2(1–3)^ab^	0.042	1(1–2)	2(1–3)	2(1–3)	0.144
Lesions		11 (100%)	26 (100%)	14 (100%)	-	21 (80.8%)	19 (100%)	10 (100%)	0.083
Lesion localization	optic nerve	3 (27.3%)	10 (38.5%)	4 (28.6%)	0.790	12 (46.2%)	8 (42.1%)	6 (60%)	0.648
	bilat ON	2 (18.2%)	6 (23.1%)	6 (42.9%)	0.357	1 (3.8%)	1 (5.3%)	1 (10%)	0.765
	cervical	6 (54.5%)	16 (61.5%)	5 (35.7%)	0.294	10 (38.5%)	14 (73.7%)	4 (40%)	0.052
	thoracic	5 (45.5%)	15 (57.7%)	11 (78.6%)	0.218	4 (15.4%)^a^	10 (52.6%)^b^	2 (20%)^ab^	0.020
	Conus	0 (0%)	1 (3.8%)	1 (7.1%)	1.000	0 (0%)	1 (5.3%)	0 (0%)	0.527
	area postrema	2 (18.2%)	3 (11.5%)	1 (7.1%)	0.728	2 (7.7%)	0 (0%)	1 (10%)	0.407
	brainstem	1 (9.1%)	6 (23.1%)	2 (14.3%)	0.707	3 (11.5%)	2 (10.5%)	2 (20%)	0.750
	cortical	2 (18.2%)	3 (11.5%)	1 (7.1%)	0.728	0 (0%)	1 (5.3%)	1 (10%)	0.273
	subcortical	0 (0%)	0 (0%)	0 (0%)	-	0 (0%)	1 (5.3%)	1 (10%)	0.273
Spinal cord lesion length		4.5(2–6)	4(2–7)	4(2–7)	0.892	0(0–2)^a^	2(2–5)^b^	0.5(0–2)^ab^	0.007
EDSS at nadir EDSS last		3(2–6)^a^ 1(1–2)^a^	6(4–8)^b^ 3(2–3)^b^	5(3–7)^b^ 3(2–3)^b^	0.001 0.002	2(0–4)^a^ 0(0–2)^a^	6(3–8)^b^ 2(1–5)^b^	4(1–6)^b^ 1(0–2)^ab^	0.002 0.005
Attack Treatment	Puls steroid	8 (72.7%)	21 (80.8%)	6 (42.9%)	0.054	22 (84.6%)	16 (84.2%)	10 (100%)	0.651
	plasma exchange	1 (9.1%)	5 (19.2%)	1 (7.1%)	0.569	2 (7.7%)	3 (15.8%)	0 (0%)	0.570
	Alternative days: Puls KS/Plasma exchange	3 (27.3%)	12 (46.2%)	6 (42.9%)	0.560	4 (15.4%)	2 (10.5%)	0 (0%)	0.640
	others	0 (0%)	0 (0%)	1 (7.1%)	0.490	1 (3.8%)	1 (5.3%)	0 (0%)	1.000
Current Immunotherapies		11 (100%)	26 (100%)	13 (92.9%)	0.490	15 (57.7%)	17 (89.5%)	7 (70%)	0.068
	Corticosteroids	0 (0%)	1 (3.8%)	0 (0%)	1.000	5 (19.2%)	4 (21.1%)	1 (10%)	0.813
	Azathioprine	7 (63.6%)	6 (23.1%)	5 (35.7%)	0.059	10 (38.5%)	9 (47.4%)	6 (60%)	0.498
	Rituximab	4 (36.4%)	14 (53.8%)	8 (57.1%)	0.538	3 (11.5%)	5 (26.3%)	2 (20%)	0.470
	Endoxan	0 (0%)	0 (0%)	0 (0%)	-	0 (0%)	0 (0%)	0 (0%)	-
	Eculizumab	0 (0%)	4 (15.4%)	1 (7.1%)	0.484	0 (0%)	0 (0%)	0 (0%)	-
	MMF	0 (0%)	1 (3.8%)	0 (0%)	1.000	0 (0%)	0 (0%)	0 (0%)	-
	Ivıg	0 (0%)	1 (3.8%)	0 (0%)	1.000	0 (0%)	0 (0%)	0 (0%)	-
Number of previous Immunotherapies		1(0–1)	1(0–2)	1(0–1)	0.596	0(0–0)	0(0–1)	0.5(0–1)	0.132
Comorbidity	No comorbidity	6 (54.5%)	14 (53.8%)	8 (57.1%)	0.896	24 (92.3%)	15 (78.9%)	8 (80%)	0.310
	Comorbidities	3 (27.3%)	10 (38.5%)	5 (35.7%)		2 (7.7%)	2 (10.5%)	2 (20%)	
	Autoimmune comorbidities	2 (18.2%)	2 (7.7%)	1 (7.1%)		0 (0%)	2 (10.5%)	0 (0%)	
S-LANSS score		0(0–3)^a^	16.5(13–19)^b^	8.5(6–11)^a^	< 0.001	2(0–3)^a^	15(10–18)^b^	4(3–9)^a^	< 0.001
NRS (Pain intensity)		0(0–0)^a^	6(4–8)^b^	4(2–7)^b^	< 0.001	0(0–1)^a^	7(6–9)^b^	2.5(0–5)^a^	< 0.001
Spasticity-related pain		0 (0%)	3 (11.5%)	1 (7.1%)	0.800	0 (0%)	0 (0%)	0 (0%)	-
Painful tonic spasms		0 (0%)^ab^	0 (0%)^b^	3 (21.4%)^a^	0.025	0 (0%)	0 (0%)	0 (0%)	-
Lhermitte’s sign		0 (0%)^a^	11 (42.3%)^b^	0 (0%)^a^	< 0.001	0 (0%)^a^	6 (31.6%)^b^	0 (0%)^ab^	0.001
Distal lower extremities neuropathic pain		0 (0%)^a^	17 (65.4%)^b^	0 (0%)^a^	< 0.001	0 (0%)^a^	12 (63.2%)^b^	0 (0%)^a^	< 0.001
Trigeminal nevralgia		0 (0%)	1 (3.8%)	0 (0%)	1.000	0 (0%)	0 (0%)	0 (0%)	-
Patients without prophylaxis for pain		1 (9.1%)^a^	22 (84.6%)^b^	9 (64.3%)^b^	< 0.001	0 (0%)^a^	17 (89.5%)^b^	2 (20%)^a^	< 0.001
Prophylaxis for pain									
	gabapentin	1 (9.1%)	6 (23.1%)	2 (14.3%)	0.707	0 (0%) ^a^	15 (78.9%) ^b^	0 (0%) ^a^	< 0.001
	pregabalin	0 (0%)^a^	12 (46.2%)^b^	4 (28.6%)^ab^	0.013	0 (0%)	2 (10.5%)	0 (0%)	0.145
	SNRI	0 (0%)^a^	10 (38.5%)^b^	4 (28.6%)^ab^	0.041	0 (0%)	1 (5.3%)	2 (20%)	0.052
	Carbamazepine	0 (0%)	4 (15.4%)	0 (0%)	0.165	0 (0%)	0 (0%)	0 (0%)	-
	Oxcarbazepine	0 (0%)	1 (3.8%)	0 (0%)	1.000	0 (0%)	0 (0%)	0 (0%)	-
	TCA:6	0 (0%)	0 (0%)	1 (7.1%)	0.490	0 (0%)	0 (0%)	0 (0%)	-
	baclofen oral	0 (0%)	2 (7.7%)	0 (0%)	0.715	0 (0%)	0 (0%)	0 (0%)	-
	baclofen pump	0 (0%)	1 (3.8%)	1 (7.1%)	1.000	0 (0%)	0 (0%)	0 (0%)	-
Number of previous pain treatment		0(0–0)^a^	1(0–2)^b^	1(0–2)^b^	0.002	0(0–0)^a^	0(0–0)^b^	0(0–0)^ab^	0.018
HAD-Anxiety score		5(3–7)	8.5(5–11)	7(4–10)	0.200	3.5(1–5)^a^	12(5–14)^b^	8(2–10)^ab^	< 0.001
HAD- depression score		5(2–9)	8(6–11)	7(6–9)	0.391	2(0–5)^a^	8(3–12)^b^	8(4–9)^ab^	0.001
PSQI Scores		4(2–7)	7(6–12)	6.5(5–8)	0.054	5(3–7)^a^	9(7–12)^b^	8.5(6–11)^ab^	0.002
SF-36: Physical Functioning Score		85(55–90)	70(40–90)	85(40–90)	0.647	90(85–95)^a^	50(30–70)^b^	67.5(55–80)^b^	< 0.001
SF-36: Role limitations due to physical health		100(0-100)	50(0–50)	25(0–50)	0.175	100(75–100)^a^	25(0–50)^b^	25(25–75)^ab^	0.003
SF-36: Role limitations due to emotional problems		100(33.33–100)	33.33(33–66)	33.315(0-100)	0.295	100(100–100)^a^	33(0–66)^b^	49.5(0-100)^b^	< 0.001
SF-36: Energy/fatigue		55(40–75)	40(25–60)	47.5(25–65)	0.181	60(60–70)^a^	50(35–60)^b^	45(35–55)^b^	0.004
SF-36: Emotional well-being		68(64–88)	66(40–84)	64(56–72)	0.493	74(68–88)^a^	56(44–64)^b^	48(32–64)^b^	< 0.001
SF-36: Social functioning		75(62.5–75) ^a^	50(25–75) ^b^	62.5(50–75) ^ab^	0.030	81.25(75–100)^a^	37.5(25–75)^b^	50(25–75)^ab^	0.004
SF-36: Pain		90(57.5–100) ^a^	45(35-77.5) ^b^	40(35-57.5) ^b^	0.002	90(55–90)^a^	35(22.5–45)^b^	50(40-57.5)^b^	< 0.001
General Health		55(37.5–80)	35(25–60)	45(35–65)	0.167	65(30–80)^a^	35(25–50)^b^	30(20–55)^ab^	0.010

Descriptive statistics are presented as medians (IQRs) or frequencies with percentages (%)

Pulse steroid therapy was commonly used across groups. Among patients with neuropathic pain, 80.8% of the NMOSD patients and 84.2% of the MOGAD patients received pulse steroids (*p* = 1.000), (Table 1).

### Comorbidities, pain intensity and prophylaxis

Comorbidities were more common in NMOSD patients (38.5%) than in MOGAD patients (10.5%), but the difference was not significant (*p* = 0.099). In the NMOSD patient group, pain intensity, as measured by the NRS, was significantly greater in the “neuropathic pain” group (median 6 [4–8]) than in the “without pain” group (median 0 [0–0]) (*p* < 0.001). Similarly, the S-LANSS score was significantly greater in the neuropathic pain group (*p* < 0.001), (Table 1).

In the MOGAD group, the NRS scores were significantly greater in the “neuropathic pain” group (median 7 [6–9]) than in the “nonneuropathic pain” group (median 2.5 [0–5]) (*p* < 0.001). The median S-LANSS scores were also significantly greater in the neuropathic pain group (*p* < 0.001). A total of 89.5% of patients in the “neuropathic pain” group received no prophylaxis, whereas 20% of patients in the “nonneuropathic pain” group received no prophylaxis (*p* < 0.001), (Table 1).

In the NMOSD group, 42.3% of patients who experienced neuropathic pain presented with Lhermitte’s sign, whereas 65.4% reported neuropathic pain in the distal lower extremities. This finding was statistically significant compared with that of the “nonneuropathic pain” group (*p* < 0.001). Additionally, 21.4% of patients in the “nonneuropathic pain” group experienced painful tonic spasms, which significantly differed from those in the “neuropathic pain” group (*p* = 0.025). In the NMOSD cohort, 84.6% of patients with neuropathic pain and 64.3% with nonneuropathic pain did not receive prophylaxis, with no significant difference in prophylaxis use between the two groups (Table 1).

### Quality of life measures

In the MOGAD, the neuropathic pain group reported lower SF-36 scores for physical functioning (*p* < 0.001), role limitations (*p* = 0.003), emotional well-being, fatigue, and general health (*p* < 0.010). Neuropathic pain was associated with lower social functioning and pain scores in both the NMOSD (*p* = 0.030, *p* = 0.002) and MOGAD (*p* = 0.004, *p* < 0.001) patients (Table 1).

### Anxiety, depression and sleep quality

Compared with patients in the “without pain” group, those in the MOGAD group had higher anxiety and depression scores (*p* < 0.001, *p* = 0.001) and poorer sleep quality (PSQI score 9 vs. 5; *p* = 0.002), (Table 1).

### Comparison of patients with neuropathic pain: NMOSD vs. MOGAD

Among the patients with neuropathic pain, 84.6% with NMOSD and 68.4% with MOGAD were female (*p* = 0.281). NMOSD patients were older, with a median age of 48.5 years, than MOGAD patients, with a median age of 38 years (*p* = 0.035). Smoking habits and disease duration were similar between the groups (*p* = 0.529, *p* = 0.790).

Optic neuritis and transverse myelitis were common first presentations in both groups, but the differences were not significant (*p* > 0.05). Brainstem involvement was more common in NMOSD patients (15.4%) than in MOGAD patients (5.3%), although the difference was not statistically significant (*p* = 0.378). Lesion localization was consistent across both groups, with cervical lesions observed in 61.5% of the NMOSD patients and 73.7% of the MOGAD patients (*p* = 0.393). Lesion length tended to be greater in the NMOSD patients than in the HCs but was not significant (*p* = 0.151).

NMOSD patients had slightly higher EDSS scores (median 3 vs. 2), but the difference was not significant (*p* = 0.283). Pulse steroids were used similarly in both groups (*p* = 1.000), but a significantly greater proportion of NMOSD patients received alternative day pulse corticosteroid and plasma exchange therapy than MOGAD patients did (46.2% vs. 10.5%, *p* = 0.011). The NMOSD neuropathic pain group had a greater median number of previous immunotherapies (1 [0–2]) than did the MOGAD group (0 [0–1]), with a significant difference (*p* = 0.014).

The incidence of neuropathic pain was comparable between the NMOSD (92.3%) and MOGAD (94.7%) groups on the basis of the S-LANSS scores (*p* = 1.000). Compared with the MOGAD group, the NMOSD group received more previous pain prophylactic treatments (*p* < 0.001). Gabapentin was more frequently used in MOGAD patients (78.9%) than in NMOSD patients (23.1%; *p* < 0.001), whereas NMOSD patients more often used pregabalin (*p* = 0.011) and SNRIs (*p* = 0.014).

**Fig. 2:**
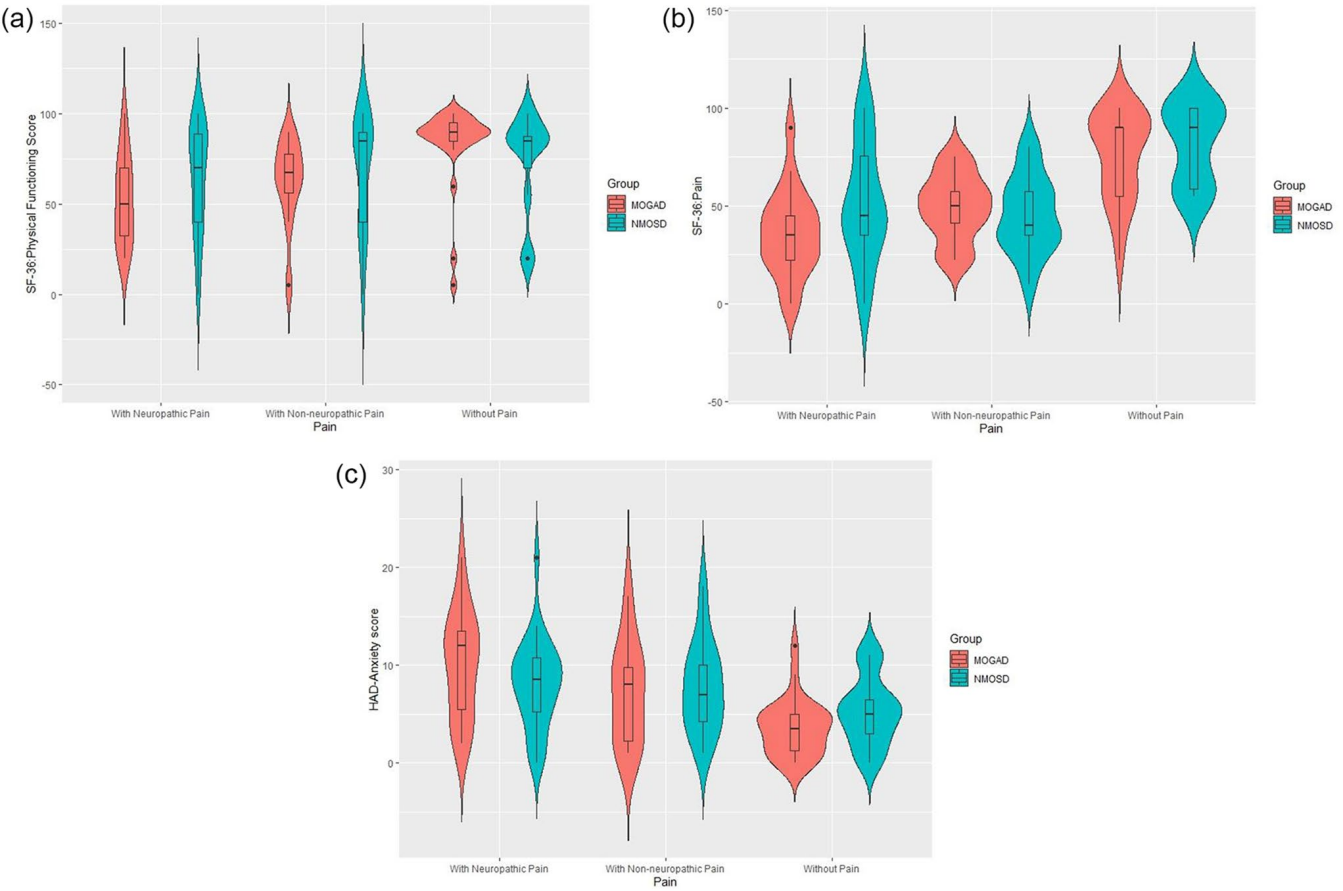
**a**, **b**, **c**, Comparison of SF-36 subdomain scores between the NMOSD and MOGAD patient groups

Comorbidities were more common in the NMOSD patients (38.5%) than in the MOGAD patients (10.5%), although the difference was not significant (*p* = 0.099). NRS scores and SF-36 quality-of-life scores were not significantly different between the groups (*p* > 0.05) (Fig. 2a and b, and 2c).

**Fig. 3:**
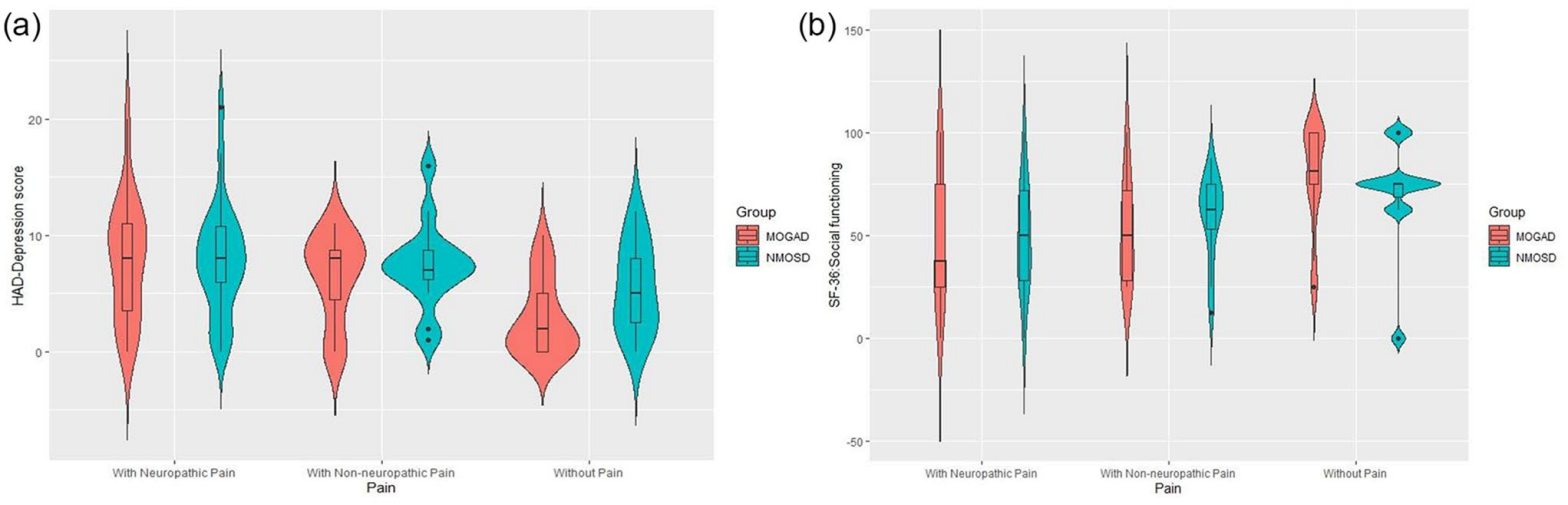
**a**, **b**, Comparison of the HAD-Anxiety and HAD-Depression scores between the NMOSD and MOGAD patient groups

Anxiety, depression, and sleep quality were similar between NMOSD patients and MOGAD patients with neuropathic pain, with no significant differences in HADS or PSQI scores (*p* > 0.05) (Table 2; Fig. 3a and b).

**Table 2:** Comparison of NMOSD and MOGAD patients with neuropathic pain

Neuropathic Pain		NMOSD (*n* = 26)	MOGAD (*n* = 19)	*p* value
*Gender*	Famale	22 (84.6%)	13 (68.4%)	0.281
	Male	4 (15.4%)	6 (31.6%)	
*Age*		48.5(42–55)	38(27–46)	0.035
*Smoking*	Active smoker	7 (26.9%)	7 (36.8%)	0.529
	Ex-smoker	5 (19.2%)	5 (26.3%)	
	Never used	14 (53.8%)	7 (36.8%)	
*Disease duration/year*		4(3–6)	4(3–6)	0.790
*First Clinical Presentation*	Optic neuritis	11 (42.3%)	10 (52.6%)	0.493
	Transverse myelitis	15 (57.7%)	11 (57.9%)	0.989
	Area postrema	1 (3.8%)	1 (5.3%)	1.000
	Brainstem	4 (15.4%)	1 (5.3%)	0.378
	Cerebral	0 (0%)	0 (0%)	-
	Others	0 (0%)	0 (0%)	-
*Number of relapses*		2(1–5)	2(1–3)	0.257
*Lesion*		26 (100%)	19 (100%)	-
*Lesion localization*	Optic nerve	10 (38.5%)	8 (42.1%)	0.805
	Bilat ON	6 (23.1%)	1 (5.3%)	0.211
	Cervical	16 (61.5%)	14 (73.7%)	0.393
	Thoracal	15 (57.7%)	10 (52.6%)	0.736
	Conus	1 (3.8%)	1 (5.3%)	1.000
	Area postrema	3 (11.5%)	0 (0%)	0.252
	Brainstem	6 (23.1%)	2 (10.5%)	0.435
	Cortical	3 (11.5%)	1 (5.3%)	0.627
	Subcortical	0 (0%)	1 (5.3%)	0.422
*Spinal Lesion length*		4(2–7)	2(2–5)	0.151
*EDSS last*		3(2–3)	2(1–5)	0.283
*Attack Treatment*	Puls steroid	21 (80.8%)	16 (84.2%)	1.000
	plasma exchange	5 (19.2%)	3 (15.8%)	1.000
	Alternative days Puls corticosteroids/Plasma exchange	12 (46.2%)	2 (10.5%)	0.011
	Others	0 (0%)	1 (5.3%)	0.422
*Current Immunotherapies*		26 (100%)	17 (89.5%)	0.173
	Corticosteroids	1 (3.8%)	4 (21.1%)	0.146
	Azathioprine	6 (23.1%)	9 (47.4%)	0.088
	Rituximab	14 (53.8%)	5 (26.3%)	0.065
	Endoxan	0 (0%)	0 (0%)	-
	Eculizumab	4 (15.4%)	0 (0%)	0.126
	MMF	1 (3.8%)	0 (0%)	1.000
	Ivıg	1 (3.8%)	0 (0%)	1.000
*Number of previous Immunotherapies*		1(0–2)	0(0–1)	0.014
*Comorbidities*	No comorbidity	14 (53.8%)	15 (78.9%)	0.099
	Comorbidities	10 (38.5%)	2 (10.5%)	
	Autoimmune comorbidities	2 (7.7%)	2 (10.5%)	
*Neuropathic pain according to S-LANSS*		24 (92.3%)	18 (94.7%)	1.000
*S-LANSS score*		16.5(13–19)	15(10–18)	0.433
*NRS (Pain intensity)*		6(4–8)	7(6–9)	0.217
*Spasticity-related pain*		3 (11.5%)	0 (0%)	0.252
*Painful tonic spasms*		0 (0%)	0 (0%)	-
*Lhermitte’s sign*		11 (42.3%)	6 (31.6%)	0.463
*Distal lower extremities neuropathic pain*		17 (65.4%)	12 (63.2%)	0.878
*Trigeminal nevralgia*		1 (3.8%)	0 (0%)	1.000
*Patients without prophylaxis for pain Prophylaxis for pain*		22 (84.6%)	17 (89.5%)	1.000
	Gabapentin	6 (23.1%)	15 (78.9%)	< 0.001
	Pregabalin	12 (46.2%)	2 (10.5%)	0.011
	SNRI	10 (38.5%)	1 (5.3%)	0.014
	Carbamazepine	4 (15.4%)	0 (0%)	0.126
	Oxcarbazepine	1 (3.8%)	0 (0%)	1.000
	TCA:6	0 (0%)	0 (0%)	-
	baclofen oral	2 (7.7%)	0 (0%)	0.501
	baclofen pump	1 (3.8%)	0 (0%)	1.000
*Number of previous pain treatment*		1(0–2)	0(0–0)	< 0.001
*HAD-Anxiety score*		8.5(5–11)	12(5–14)	0.160
*HAD- depression score*		8(6–11)	8(3–12)	0.818
*PSQI Scores*		7(6–12)	9(7–12)	0.321
*SF-36: Physical Functioning Score*		70(40–90)	50(30–70)	0.107
*SF-36: Role limitations due to physical health*		50(0–50)	25(0–50)	0.794
*SF-36: Role limitations due to emotional problems*		33.33(33–66)	33(0–66)	0.406
*SF-36: Energy/fatigue*		40(25–60)	50(35–60)	0.374
*SF-36: Emotional well-being*		66(40–84)	56(44–64)	0.084
*SF-36: Social functioning*		50(25–75)	37.5(25–75)	0.684
*SF-36: Pain*		45(35-77.5)	35(22.5–45)	0.079
*General Health*		35(25–60)	35(25–50)	0.817

Descriptive statistics are presented as medians (IQRs) or frequencies with percentages (%)

## Discussion

Our study explored demographic, clinical, and radiological predictors of chronic neuropathic pain in patients with NMOSD and MOGAD, along with their impact on sleep and quality of life. The prevalence of pain was greater in patients with NMOSD (78.4%) than in those with MOGAD (52.7%), likely due to differences in pathology and lesion localization. Neuropathic pain was diagnosed in 50.9% of the NMOSD patients and 34.5% of the MOGAD patients. These findings align with those of Asseyer et al.., who reported higher neuropathic pain rates in patients with NMOSD [11]. However, our study adds to this understanding by highlighting a slightly lower but still significant prevalence of neuropathic pain in MOGAD patients (34.5%). Although Xue et al.. reported similar pain levels across both diseases, our study revealed slightly greater pain intensity in MOGAD (median NRS score of 7) than in NMOSD (median NRS score of 6), although the difference was not statistically significant [7].

Neuropathic pain was more commonly associated with longer spinal cord lesions, particularly in MOGAD patients. In addition, MOGAD patients with neuropathic pain had poorer sleep quality and significantly lower quality of life scores, as assessed by the SF-36. Another important point is that patients with neuropathic pain reported higher anxiety and depression scores, especially in the MOGAD group, which further contributed to their impaired quality of life.

We also observed significant differences between NMOSD patients and MOGAD patients in terms of pain management strategies. NMOSD patients were more likely to receive combination therapies, including pregabalin and SNRIs, whereas MOGAD patients were more frequently treated with gabapentin monotherapy. Despite these interventions, a large proportion of patients, especially those in the NMOSD group, did not receive adequate pain prevention therapy. Earlier studies reported suboptimal pain control in NMOSD and MOGAD patients [10]. Similar to our study results, the high proportion of patients without prophylactic treatment for pain, particularly in the NMOSD group, highlights the unmet need for better pain management protocols.

Overall, our findings emphasize the substantial burden that neuropathic pain places on NMOSD and MOGAD patients, which negatively affects their physical, mental, and emotional well-being. These results underscore the necessity for more personalized and proactive approaches to managing pain in these patient groups.

One of the key findings of our study is the strong association between lesion characteristics and neuropathic pain, particularly in MOGAD patients. We observed that patients with longer spinal cord lesions, particularly in the thoracic region, were more likely to experience neuropathic pain. This association was especially pronounced in the MOGAD group, where a significantly greater frequency of thoracic lesions was observed in patients with neuropathic pain. The localization of lesions in the spinal cord, especially in the thoracic and cervical regions, likely plays a crucial role in the development of neuropathic pain by disrupting the sensory pathways involved in pain processing. Previous studies have similarly reported the impact of lesion length and location on pain outcomes in both NMOSD patients and MOGAD patients [4, 11]. Our findings extend this understanding by reinforcing the role of spinal cord lesion characteristics in predicting pain severity, particularly in MOGAD. These results suggest that lesion characteristics should be routinely considered in the clinical evaluation and management of pain in NMOSD and MOGAD patients, as they could serve as important indicators for the development of chronic pain.

Our study revealed a significant correlation between the frequency of relapses and the development of neuropathic pain, particularly in NMOSD patients. Patients with more frequent attacks tended to experience more severe neuropathic pain. This observation supports the hypothesis that recurrent inflammatory episodes in NMOSD exacerbate nerve damage, leading to heightened pain sensitivity and chronic pain development. These findings align with those of previous studies, such as Zhang et al.., which also demonstrated that frequent relapses in NMOSD patients contribute to greater pain severity [8]. In contrast, MOGAD patients did not show a similar correlation between the number of attacks and pain severity. These findings suggest that the mechanisms underlying neuropathic pain in MOGAD patients may differ from those in NMOSD patients. In MOGAD, pain development may be more closely linked to specific lesion characteristics, such as location and extent, rather than the number of relapses. This distinction highlights the need for tailored pain management approaches that consider disease pathology and progression differences. The relationship between relapses and pain in NMOSD further underscores the importance of relapse prevention and aggressive disease-modifying therapies in managing chronic pain in this population. Reducing the frequency of attacks may mitigate the development of neuropathic pain and improve long-term outcomes for NMOSD patients.

Interestingly, transverse myelitis was a more frequent initial presentation in patients with neuropathic pain in both NMOSD and MOGAD, corroborating findings by Valerio et al.. that linked transverse myelitis with a greater likelihood of chronic pain development. In our MOGAD cohort, the prevalence of thoracic spinal cord lesions was significantly greater in patients with neuropathic pain, further supporting the role of lesion location in the development of pain, as suggested by earlier studies [6]. These findings suggest that transverse myelitis and the resulting spinal cord lesions may serve as predictors for the development of chronic pain in both NMOSD and MOGAD patients. The association between transverse myelitis and neuropathic pain highlights the importance of early intervention and aggressive management during the initial presentation of transverse myelitis. Prompt treatment aimed at reducing inflammation and preventing further spinal cord damage could reduce the risk of developing chronic pain in these patients.

Patients experiencing neuropathic pain reported significantly lower scores in multiple quality-of-life domains, including physical functioning, role limitations, and emotional well-being. These findings are in line with those of Mealy et al.., who reported that chronic pain in NMOSD patients led to profound limitations in daily functioning and reduced overall quality of life [5]. Our data extend this understanding to MOGAD patients, who similarly demonstrated impaired physical and emotional functioning in the presence of neuropathic pain.

Our study highlighted the significant impact of neuropathic pain on sleep quality in both NMOSD patients and MOGAD patients. Those suffering from neuropathic pain reported higher scores on the PSQI, indicating poorer sleep quality than patients without pain. This was particularly evident in the MOGAD group, where patients with neuropathic pain had significantly worse sleep quality than those without pain. This finding is consistent with the work of Ju et al.., who highlighted the psychological burden of pain in patients with NMOSD and MOGAD [3]. Chronic pain is well known to disrupt sleep, and poor sleep, in turn, can exacerbate pain perception, creating a vicious cycle. This link between neuropathic pain and impaired sleep quality emphasizes the need to address sleep disturbances as part of a comprehensive pain management strategy. Poor sleep not only worsens the pain experience but also negatively affects patients’ overall quality of life, cognitive function, and emotional well-being. The relationship between pain and sleep disturbances should prompt clinicians to incorporate sleep assessments into routine care for NMOSD and MOGAD patients experiencing chronic pain. Interventions aimed at improving sleep quality, such as cognitive‒behavioral therapy for insomnia (CBT-I) or pharmacological treatments tailored to pain and sleep.

Our study has several limitations. The cross-sectional design limits our ability to determine causal relationships between clinical characteristics, lesion findings, and neuropathic pain. A longitudinal design would better capture the progression of pain and its link to disease activity. Additionally, the small sample size, especially in the MOGAD group, may have reduced statistical power. Larger, more diverse cohorts are needed for more robust analyses. Recall bias is another limitation, as patient-reported outcomes may not accurately reflect experiences over time; objective measures such as polysomnography could provide more precise data. Finally, while we identified correlations between lesions and pain, this study did not explore the underlying pathophysiological mechanisms, including the role of inflammatory mediators, nerve fiber damage, and central pain processing pathways, which future research should address to develop targeted treatments.

In conclusion, this study highlights the significant burden of neuropathic pain in patients with NMOSD and MOGAD, demonstrating its profound impact on quality of life, psychological well-being, and daily functioning. Neuropathic pain is closely associated with reduced physical function, poorer sleep quality, and higher levels of anxiety and depression, further exacerbating the challenges faced by these patients. Despite advancements in the treatment of NMOSD and MOGAD, pain management remains suboptimal, with many patients not receiving adequate prophylaxis or relief from current pain management strategies. This underscores the urgent need for more personalized and comprehensive approaches to pain management, which consider both the physical and psychological dimensions of chronic pain. Moving forward, future research should focus on longitudinal studies to establish causal relationships and explore the underlying mechanisms of neuropathic pain. Additionally, developing more effective and targeted pain management protocols incorporating both pharmacological and nonpharmacological approaches will be crucial to improving outcomes for patients with NMOSD and MOGAD.

## Data Availability

The datasets used and/or analyzed during the current study are available from the corresponding author upon reasonable request.
